# Reversal of National Coverage Determination for MR Spectroscopy Increases Reimbursement Rates

**DOI:** 10.1148/rycan.240230

**Published:** 2024-08-30

**Authors:** Candace C. Fleischer, Alexander P. Lin, Jason W. Allen

**Affiliations:** Department of Radiology and Imaging Sciences, Emory University School of Medicine, 1750 Haygood Dr, Atlanta, GA 30322*; Center for Clinical Spectroscopy, Department of Radiology, Brigham and Women’s Hospital, Boston, Mass^†^; Department of Radiology and Imaging Sciences, Indiana University School of Medicine, Indianapolis, Ind^‡^

## Editor:

In a recent review article describing technical advances toward routine and regular integration of MR spectroscopy in clinical cancer management (1), Dr Fleischer, Dr Allen, and colleagues discuss the recent retirement of the national coverage determination (NCD) for MR spectroscopy. The authors note the potential financial burden on patients when MR spectroscopy is performed; however, this is only true in some cases. As lack of insurance coverage has been a key barrier for clinical MR spectroscopy, we would like to clarify the potential impact of this NCD retirement.

## CPT Code for MR Spectroscopy

There has been an established current procedural terminology (CPT) code for MR spectroscopy, 76390, since 1998. Reimbursement rates have existed for both Centers for Medicare and Medicaid (CMS) and private insurance payers for this code; however, in 2002, an NCD for MR spectroscopy was put in place by CMS (2). This NCD limited coverage for MR spectroscopy for all indications, effectively resulting in underutilization of clinical MR spectroscopy. In 2021, this NCD for noncoverage was retired and, as a result, coverage determination is now made by regional Medicare administrative contractors (MACs).

As of May 2024, at least six MACs have reimbursed for MR spectroscopy, including Novitas (covering Texas and neighboring states), Palmetto (Southeast), CGS (Ohio and Kentucky), First Coast (Florida, Puerto Rico, and U.S. Virgin Islands), Noridian (West Coast and Northwest), and NGS (Midwest and Northeast), reimbursing up to $507.17 per MR spectroscopy scan (3). Several private payers also currently reimburse for MR spectroscopy for specific indications, including Aetna, Cigna, and Blue Cross/Blue Shield (4), and are billed up to $3928 per MR spectroscopic examination (5). Based on our anecdotal experience, however, this CPT code is likely being underbilled due to lack of awareness of this change in NCD.

## Clinical Use of MR Spectroscopy

Overall, retirement of the NCD, as it limited coverage, is seen as an important step toward increased clinical usage of MR spectroscopy. We believe it is important for clinical practice groups to be aware of this change and encourage the appropriate use and billing of MR spectroscopy.
